# Retraction Note: Chitosan hydrogel/silk fibroin/Mg(OH)_2_ nanobiocomposite as a novel scaffold with antimicrobial activity and improved mechanical properties

**DOI:** 10.1038/s41598-025-88190-2

**Published:** 2025-02-11

**Authors:** Reza Eivazzadeh-Keihan, Fateme Radinekiyan, Hooman Aghamirza Moghim Aliabadi, Sima Sukhtezari, Behnam Tahmasebi, Ali Maleki, Hamid Madanchi

**Affiliations:** 1 Catalysts and Organic Synthesis Research Laboratory, Department of Chemistry, Iran University of Science and Technology, 16846-13114 Tehran, Iran; 2 Faculty of Chemistry, K.N. Toosi University of Technology, Tehran, Iran; 3 Protein Chemistry Laboratory, Department of Medical Biotechnology, Biotechnology Research Center, Pasteur Institute of Iran, Tehran, Iran; 4 School of Chemistry, College of Science, University of Tehran, Tehran, Iran; 5 Department of Biotechnology, School of Medicine, Semnan University of Medical Sciences, Semnan, Iran; 6 Drug Design and Bioinformatics Unit, Department of Medical Biotechnology, Biotechnology Research Center, Pasteur Institute of Iran, Tehran, Iran

Retraction of: *Scientific Reports* 10.1038/s41598-020-80133-3, published online 12 January 2021

Editors have retracted this Article.

After publication of this Article, concerns about the data were brought to the attention of the Editors. Specifically, in Figure 7c the image panels for Control and CTT-CS/SF/Mg(OH)2 nanobiocomposite appear to overlap. The Editors requested that the Authors provide full raw data and copies of their ethics approval and protocol for the use of blood samples drawn from a human donor, but the Authors were not able to do so. The Editors no longer have confidence in the reliability of the results and findings presented in this Article.

Hamid Madanchi disagrees with this retraction. The remaining authors did not respond to correspondence from the Editors regarding this retraction.

